# Phylogeographic Diversity of Pathogenic and Non-Pathogenic Hantaviruses in Slovenia

**DOI:** 10.3390/v5123071

**Published:** 2013-12-10

**Authors:** Miša Korva, Nataša Knap, Katarina Resman Rus, Luka Fajs, Gašper Grubelnik, Matejka Bremec, Tea Knapič, Tomi Trilar, Tatjana Avšič Županc

**Affiliations:** 1 Institute of Microbiology and Immunology, Faculty of Medicine, University of Ljubljana, Zaloška 4, Ljubljana 1000, Slovenia; E-Mails: misa.korva@mf.uni-lj.si (M.K.); natasa.knap@mf.uni-lj.si (N.K.); katarina.resman@mf.uni-lj.si (K.R.R.); luka.fajs@mf.uni-lj.si (L.F.); grubelnik.gasper@gmail.com (G.G.); matejka.bremec@gmail.com (M.B.); 2 Slovenian Museum of Natural History, Prešernova 20, Ljubljana 1000, Slovenia; E-Mails: tea.knapic@gmail.com (T.K.); ttrilar@pms.lj.si (T.T.)

**Keywords:** hantavirus, Slovenia, epidemiology, genetic diversity, phylogeography

## Abstract

Slovenia is a very diverse country from a natural geography point of view, with many different habitats within a relatively small area, in addition to major geological and climatic differences. It is therefore not surprising that several small mammal species have been confirmed to harbour hantaviruses: *A. flavicollis* (Dobrava virus), *A. agrarius* (Dobrava virus–Kurkino), *M. glareolus* (Puumala virus), *S. areanus* (Seewis virus), *M. agrestis*, *M. arvalis* and *M. subterraneus* (Tula virus). Three of the viruses, namely the Dobrava, Dobrava–Kurkino and Puumala viruses, cause disease in humans, with significant differences in the severity of symptoms. Due to changes in haemorrhagic fever with renal syndrome cases (HFRS) epidemiology, a detailed study on phylogenetic diversity and molecular epidemiology of pathogenic and non-pathogenic hantaviruses circulating in ecologically diverse endemic regions was performed. The study presents one of the largest collections of hantavirus L, M and S sequences obtained from hosts and patients within a single country. Several genetic lineages were determined for each hantavirus species, with higher diversity among non-pathogenic compared to pathogenic viruses. For pathogenic hantaviruses, a significant geographic clustering of human- and rodent-derived sequences was confirmed. Several geographic and ecological factors were recognized as influencing and limiting the formation of endemic areas.

## 1. Introduction

In Slovenia, the first hantavirus infection was described in 1954, when a patient was infected presumably with the Puumala virus (PUUV) during forestry work in the Pohorje region [1]. Following a number of severe haemorrhagic fever with renal syndrome cases (HFRS) in the southeastern part of Slovenia, the Dobrava virus (DOBV) was isolated in 1988 from the lungs of a yellow-necked mouse captured in Dobrava village. In 1992, it was fully characterized and recognized as a unique hantavirus species [2]. Since then, epidemic outbreaks and sporadic cases have been recorded yearly, with the highest numbers recorded in 2012 (188 cases). The overall case fatality rate is 4.5% [3]. Co-existence of both DOBV and PUUV in a single endemic region has been demonstrated and it was shown that the viruses are capable of causing HFRS with significant differences in severity [3]. Initial genetic analysis of DOBV sequenced from rodents and patients revealed that DOBV in Slovenia is harboured by two distinct species of *Apodemus* mice: *A. favicollis* and *A. agrarius*. Phylogenetic analysis provided strong evidence that two distinct DOBV genotypes are present in Slovenia (Dobrava and Kurkino), but until now, only DOBV–Dobrava was detected in patients [4]. Investigation of Slovenian HFRS cases caused by PUUV has shown the existence of two distinct genetic lineages that are grouping based on their geographical origin [5]. Besides pathogenic, also non-pathogenic hantaviruses (Tula and Seewis viruses) circulate in the same zoonotic regions. High genetic diversity was detected in the Tula virus (TULV), amplified from three different *Microtus* sp. within a single endemic region. Slovenian TULV sequences showed significant geographical clustering instead of host-specific co-evolution [6]. Furthermore, three highly divergent genetic lineages of the Seewis virus (SWSV), detected in *S. araneus*, were recently reported in Slovenia. Contrary to other hantaviruses circulating in the country, SWSV does not cluster into geographic lineages, and two genetic lineages are sympatric in one study location [7].

Increasing amplitude and magnitude of HFRS outbreaks in the country and availability of diverse animal collection has enabled us to perform a detailed study on the phylogenetic diversity and molecular epidemiology of pathogenic and non-pathogenic hantaviruses.

## 2. Results and Discussion

### 2.1. Description of Zoogeographic Regions

Slovenia is formed of five zoogeographic regions: Submediterranean, Dinaric, Alpine, Prealpine and Subpannonian [8]. The formation of these regions is influenced by several abiotic factors, which influence the formation of the habitats: tectonic, lithological, relief, climatic and edaphic conditions. Tectonically, Slovenia is formed of the eastern and southern Alps, the Pannonic basin, and the Dinarides. The lithographic variability is also great, with the majority of Slovenia being rich in limestone and dolomites (Dinaric, Alpine and parts of Prealpine and Submediterranean regions), igneous rocks in the Prealpine region, slates, sandstones and limestone in the Subpannonian region and flysch in the Submediterranean part. Major rivers running through all the regions additionally influence the formation of zoogeographic areas, and are recognized as the zones of highest biological diversity and intense human activity. The Alpine region is also determined by its mountainous relief, which influences its climate and vegetation, comprised mostly of coniferous boreal type and subalpine broad-leaved forest. These forests continue into the Prealpine region, though more lowland forests, rich with beech and spruce, are evident. The Submediterranean region has a lot of influence from the Mediterranean climate, enabling the growth of oak forests, which can also be found in the Subpannonian region, where the climate is continental. The Dinaric region has many hills and sinkholes; the climate is relatively stable and humid, with microclimate areas forming due to its dynamic relief. The forests are mostly broad-leaved, with a significant presence of beech [8].

### 2.2. Collection of Animal Samples and Detection of Hantavirus RNA

Small mammals were trapped in spring and autumn, in different locations in Slovenia from 1990–2012. In total, 2,393 animals of several species—*Apodemus* sp.; *Myodes glareolus*; *Microtus* sp.; *Mus musculus*; *Glis glis*; *Arvicola terrestris*; *Crocidura* sp.; *Neomys.* sp;, and *Sorex* sp.—were trapped, and the majority of them were molecularly tested for the presence of hantaviruses. Out of 420 molecularly tested *M. glareolus*, hantavirus RNA was detected in 49 (11.6%). Molecular testing of 760 *A. favicollis* confirmed hantavirus RNA in 148 (19.5%). Out of 85 tested, *A. agrarius*, hantavirus RNA was detected in nine animals (10.6%). Furthermore, 75 other voles and shrews were captured (two *A. terrestris*, eight *M. agrestis*, 15 *M. arvalis*, five *M. nivalis*, three *M. liechtensteini*, one *M. subterraneus*, four *C. leucodon*, four *C. suaveolens*, 12 *N. anomalus*, eight *N. fodiens*, one *S. alpinus* and 12 *S. araneus*). Hantavirus RNA was confirmed in six voles (four *M. arvalis*, one *M. agrestis*, one *M. subterraneus*) and in seven common shrews (*S.araneus*)*.*

**Figure 1:**
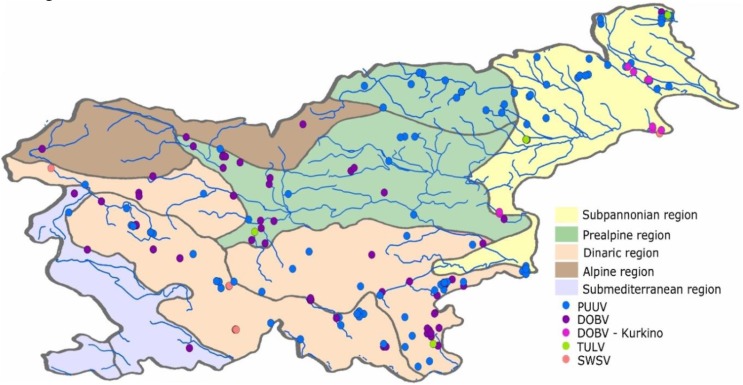
Distribution of obtained hantavirus sequences throughout zoogeographic regions of Slovenia.

For the purpose of the present study, 98 animal samples were selected from all five zoogeographic regions in Slovenia; PUUV sequences were obtained from 40 bank voles, DOBV–Dobrava sequences were obtained from 36 yellow-necked mice, DOBV–Kurkino sequences were obtained from nine striped field mice, TULV sequences from six voles and SWSV sequences from seven shrews (Figure 1).

### 2.3. Collection of Patient Samples and Detection of Hantavirus RNA

A total of 506 HFRS patients, 132 infected with DOBV and 374 infected with PUUV, have been hospitalized in Slovenia between the years 1985 and 2012. For the purpose of the study, multiplex real- time (RT-PCR) positive patients from different endemic regions were selected. PUUV sequences were obtained from 65 patients; DOBV–Dobrava sequences were amplified from 30 patients and fromDOBV–Kurkino sequences were also obtained from one patient.

### 2.4. Phylogenetic Analysis L Segment Sequences

**Figure 2:**
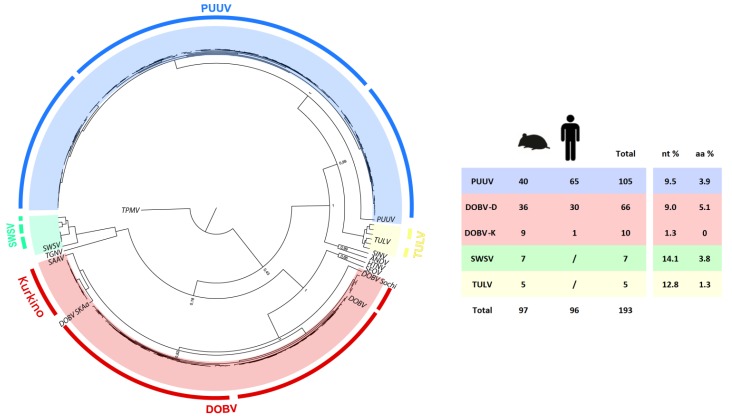
Tree representing bayesian phylogenetic analysis of L segment (235 bp) of Slovenian hantavirus genotypes, under the best-fit HYK + G model of evolution, four MCMC runs of 10,000,000 generations. The table shows the number of obtained sequences from small mammals and patients, as well as pairwise nucleotide (nt) and deduced amino acid (aa) distances.

For investigation of phylogenetic diversity of pathogenic (DOBV, DOBV–Kurkino and PUUV) and non-pathogenic (TULV and SWSV) hantaviruses present in Slovenia, partial L segment sequences were successfully amplified from 97 rodent and 96 patient samples. Bayesian phylogenetic analysis confirmed the presence of five hantavirus genotypes, PUUV, DOBV, DOBV-Kurkino, TULV and SWSV, belonging to four hantavirus species (Figure 2).

Nucleotide and deduced amino acid distances are shown in Figure 2, where the highest nucleotide diversity was observed within SWSV (14.1%) amplified from common shrews captured in three different localities. High nucleotide diversity (12.8%) was determined also in TULV amplified from three different *Microtus* sp., where sequences grouped into two phylogenetic lineages according to their geographic origin (Figure 2). In total, 105 PUUV sequences were determined, with divergence ranging from 0%–9.5% on the nucleotide level and up to 3.9% on the deduced amino acid level. Human and bank vole-derived PUUV sequences were correlated within specific geographic areas; sequences clustered into three major phylogenetic lineages ranging from the Subpannonian to the Submediterranean region (Figure 2). All DOBV–Dobrava sequences, derived only from yellow-necked mice, were closely related to each other and to DOBV sequences amplified from patients (nucleotide diversity 0%–9.0%). Above that, a clear geographic clustering showed the existence of three phylogenetic lineages. Additionally to the already established presence of DOBV–Kurkino genotype in striped field mice in the Subpannonian region, the same genotype was determined in the 1 HFRS patient from the same region. Nucleotide diversity of sequences belonging to the DOBV–Kurkino genotype was only 1.3%, with no diversity on the deduced amino acid level (Figure 2).

### 2.5. Phylogenetic Analysis of S and M Segment Sequences

**Figure 3:**
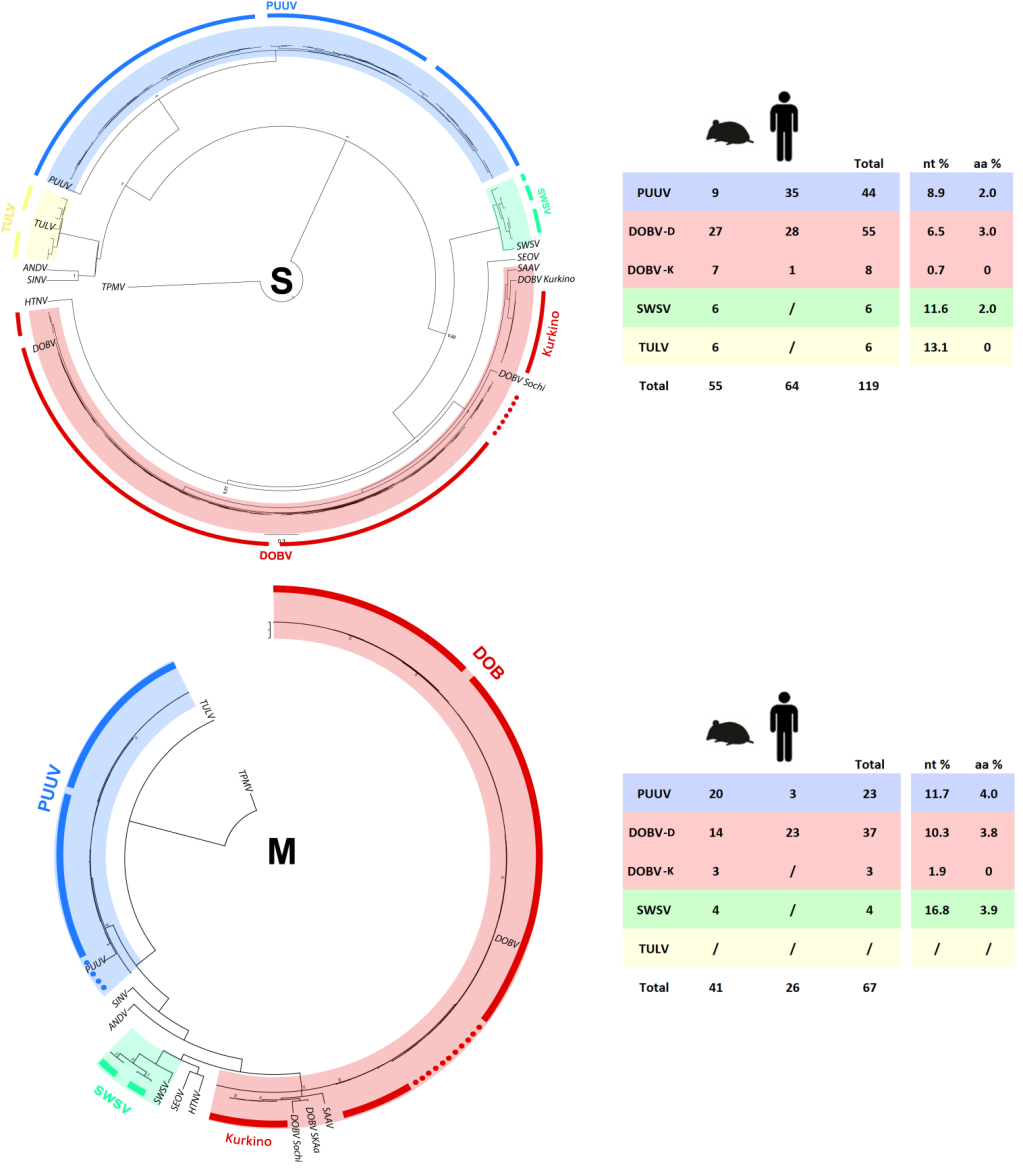
Tree representing bayesian phylogenetic analyses of S segment (306 bp) and M segment (134 bp) of Slovenian hantavirus genotypes, under the best-fit HYK + G model of evolution, four MCMC runs of 10,000,000 generations. The table shows the number of obtained sequences from small mammals and patients, as well as pairwise nucleotide (nt) and deduced amino acid (aa) distances. Dots indicate sequences that could not be assigned to any phylogenetic lineage determined in the L segment analysis.

To confirm hantavirus phylogenetic diversity, observed in the L segment, we further analysed S and M segment sequences. The phylogenetic analysis of 306 bp long S segment sequence from 119 samples, 55 rodents and 64 patients confirmed the presence of five hantavirus genotypes in Slovenia (Figure 3). Nucleotide diversity was smaller than on the L segment (Figure 2), but still SWSV (11.6%) and TULV (13.1%) have the largest nucleotide divergence observed. PUUV S segment sequences were obtained from 44 samples (9 voles and 35 patients), with diversity of 8.9% on the nucleotide level and up to 2% on the deduced amino acid level. All three phylogenetic lineages identified on the L segment sequences were confirmed (Figure 3).

DOBV S segment sequences were obtained from 55 samples (27 mice and 28 patients) and the presence of three phylogenetic lineages was confirmed. Nucleotide diversity between DOBV lineages was only 6.5%. Again, the smallest divergence was seen in the DOBV–Kurkino genotype (0.7%), where we successfully obtained sequences from seven mice and one patient sample.

Further on, we obtained 67 partial M segment sequences (134 bp) from 41 rodent and 26 patient samples. We were unsuccessful in amplifying the M segment from TULV carriers and the patient infected with the DOBV–Kurkino genotype. Due to the lower number of available sequences, we confirmed the presence of two phylogenetic lineages within PUUV, three lineages within DOBV and two SWSV genetic lineages (Figure 3). The largest divergence was observed within SWSV sequences (16.3%), whilst the smallest divergence was seen in the DOBV–Kurkino genotype (1.9%) (Figure 3).

### 2.6. Phylogeographic Analysis

**Figure 4:**
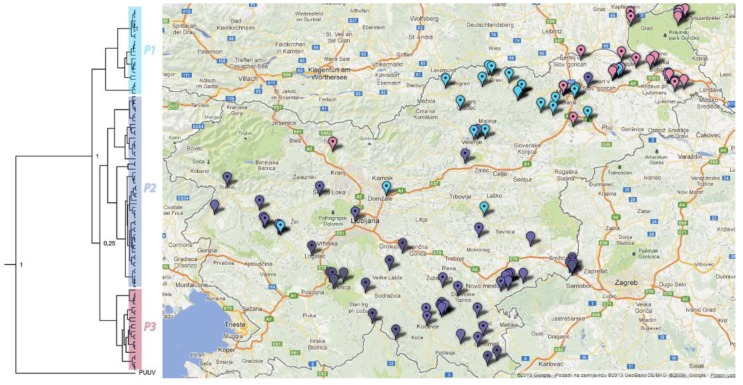
Correlation of phylogenetic and geographical clustering based on PUUV L segment sequences. Phylogeographic analysis showed the presence of three genetic lineages (light blue—P1, dark blue—P2, pink—P3) in voles and patients. The sequences obtained from patients are marked with a dot.

The PUUV and DOBV L segments were used for phylogeographic analyses due to the greatest number of acquired sequences. As seen in Figure 4, three PUUV phylogenetic lineages, designated P1–P3, are related to the geographical origin of sequences obtained from PUUV patients and hosts [7]. The first PUUV lineage (P1) is localized in the Prealpine region and the second lineage (P2) was determined mainly in patients from the Dinaric and Submediterranean regions. The majority of patients and animals infected with PUUV from lineage P3 can be found in the Subpannonian zoogeographic region Statistically significant differences were confirmed in the distribution of specific phylogenetic lineages within zoogeographic regions (χ^2^, *p* < 0.0001). Sequences obtained from bank voles and PUUV-infected patients mainly overlap and geographically outlying sequences were all derived from patients.

**Figure 5:**
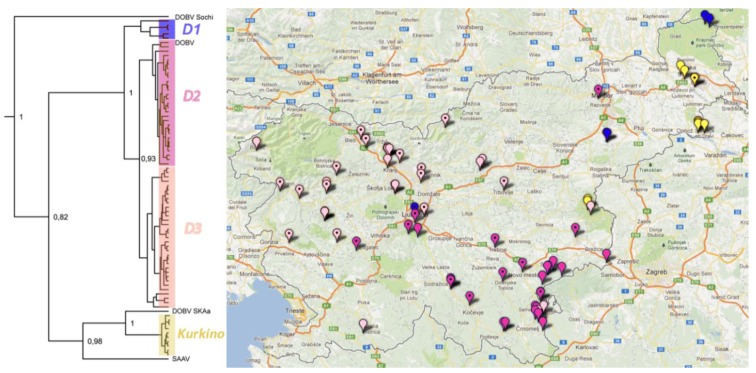
Correlation of phylogenetic and geographical clustering based on sequence of DOBV L segment. Phylogeographic analysis showed the presence of three genetic lineages of DOBV–Dobrava (violet—D1, pink—D2, orange—D3) and DOBV–Kurkino genotype in mice and patients. The sequences obtained from patients are marked with a dot.

Phylogeographic analysis of DOBV revealed three phylogenetic lineages of the DOBV–vDobrava and one lineage of the DOBV–Kurkino genotype. Distribution of specific phylogenetic lineages was correlated with zoogeographic regions (χ^2^, *p* < 0.0001). In the Subpannonian region, both DOBV lineage D1 and DOBV–Kurkino are sympatric in hosts and patients. Moreover, the DOBV–Kurkino sequence obtained from a patient clusters together with the sequences from striped field mice. Lineage DOBV D1 is circulating in a geographically limited focal area, from which patients are rarely reported. However, one sequence from lineage D1 was obtained from a patient resident in the central part of Slovenia. Phylogenetic lineages D2 and D3 show a clear geographical clustering, where sequences from mice and patients mainly overlap. Sequences from the lineage D2 are located in the Dinaric region, where DOBV was isolated for the first time and where the most severe cases of HFRS are reported. The third, the D3 lineage, is mostly found in the Prealpine and Submediterranean regions (Figure 5).

## 3. Experimental Section

### 3.1. Trapping of Small Mammals and Patient Sample Collection

Small mammals had been trapped in spring and autumn, in different locations in Slovenia since 1990. Study sites were selected with regard to data on reported HFRS cases. Sherman (Tallahassee, FL, USA) and Elliot-type (Upwey, Australia) live traps were used for the trapping of small mammals. All captured animals were identified, weighed, measured and then anesthetized. Internal organs (heart, lungs, liver, spleen, kidneys and urine bladder) were collected and stored in cryovials at −80 °C. Blood was collected by cardiac puncture and centrifuged for 5 min at 2,000 rpm. Serum was separated from the blood clot and both were stored in cryovials at −20 °C. Each caught animal was identified by a professional taxonomist and additionally confirmed by sequencing of cytochrome *b* as a phylogenetic marker (data available upon request).

Patient samples were received for HFRS testing from several Slovenian hospitals from 1985–2012. Blood was centrifuged for 5 min at 2,000 rpm. Serum was separated from the blood clot and stored at −20 °C. Clinical diagnosis was confirmed serologically by an indirect immunofluorescence assay and by enzyme-linked immunoassay IgM and IgG tests [3].

### 3.2. DNA and RNA Extraction

Total DNA was extracted by QIAamp DNA Mini Kit (Qiagen, Venlo, The Netherlands) from animal lung samples, according to the manufacturer’s instructions. Total RNA was extracted by TRIZOL Reagent (Invitrogen, Life Technologies^TM^, Carlsbad, NM, USA) from animal kidney samples, according to the manufacturer’s instructions. For total RNA extraction from human serum samples, QIAamp Viral RNA Mini Kit (Qiagen, Venlo, The Netherlands) was used, as described by the manufacturer.

### 3.3. Screening with Multiplex Real-Time RT-PCR for DOBV and PUUV

For screening of all human and animal samples from *Apodemus* sp. and *M. glareolus* multiplex real-time RT-PCR assay specific for Slovenian DOBV and PUUV was used. Multiplex real-time RT-PCR assay targeting DOBV M segment (97 bp) and PUUV S segment (186 bp) was performed using primers DOB D (ACTTTAAGACAACCAATA), DOB L (GGGCAGTGTATTTATTCAG), PUU D (GGAGTAAGCTCTTCTGC), PUU L (ACATCATTTGAGGACAT) and probes DOB S (FAM-TTCCATGGCTGGGCAACTGCT-DB) and PUU S (JOE-TTCATGCCAACAGCCCAGTCAAC-DB). Real-time RT-PCR conditions were established for ABI7500 series (Applied Biosystems, Life Technologies^TM^, Carlsbad, NM, USA) with temperature protocol 50 °C for 5 min, 95 °C for 20 s and 45 cycles consisting of 95 °C for 3 s, 55 °C for 30 s and 60 °C for 30 s. Taq Man^®^ Fast Virus 1-Step Master Mix (Applied Biosystems, Life Technologies^TM^, Carlsbad, NM, USA) was used for the reaction mix.

### 3.4. Screening with Hantavirus Universal RT-PCR

For hantavirus screening broad spectrum, nested RT-PCR with degenerated primers HAN-L-F1, HAN-L-R1, HAN-L-F2, HAN-L-R2 targeting L segment (band size 390 bp) were used [9]. For positive samples additionally S and M segments were obtained.

### 3.5. RT-PCR and Sequencing

#### 3.5.1. Dobrava Virus-Specific RT-PCR

To obtain DOBV–Dobrava S segment (band size 1700 bp) new outer primers, DOB-SF2 (ACTCCCTAAAGAGCACTACA) and DOB-SR (GGTAGTAGTTGTTGAGGT) were designed. Temperature protocol for RT-PCR was 50 °C for 30 min, 94 °C for 2 min, 40 cycles of 94 °C for 15 s, 56 °C for 30 s and 68 °C for 2 min and final extension of 68 °C for 5 min. Superscript^TM^ One-Step RT-PCR with Platinum Taq (Invitrogen, Life Technologies^TM^, Carlsbad, NM, USA) was used for the reaction mix. Additionally, nested step of the PCR (band size 1051 bp) was carried out for some samples with previously published primers MS120C and MS1170R [10].

To obtain DOBV–Dobrava M segment (band size 856 bp), new primers DOB-M1F (CAA AAT CCC ACA TAC TGC AA) and DOB-M1R (AGT CTC CCA TCA AAC CAA) were designed. Temperature protocol for RT-PCR was 50 °C for 30 min, 94 °C for 2 min, 40 cycles consisting of 94 °C for 15 s, 57 °C for 30 s and 68 °C for 1 min and final extension of 72 °C for 7 min. Superscript^TM^ One-Step RT-PCR with Platinum Taq (Invitrogen, Life Technologies^TM^, Carlsbad, NM, USA) was used for the reaction mixture.

#### 3.5.2. Dobrava-Kurkino Virus-Specific RT-PCR

For DOBV–Kurkino S segment (band size 1051 bp) from animal samples DOBV primers (DOB-SF2, DOB-SR, MS120C, MS1170R) were used. For patient samples, additional outer primers DOBS1 and DOBS2, as well as inner primers DOBS3 and DOBS4 (428 bp) were used [4]. To obtain DOBV–Kurkino M segment (band size 290 bp), previously published primers MOF 103, MOR 204, DOB G1F and DOB G1R were used [4,11].

#### 3.5.3. Puumala Virus-Specific RT-PCR

To obtain PUUV S segment (band size 1700 bp) new primers PUU-SF (AAG AGA AGA ATG GCA GA) and PUU-SR (GGU GAA AAG GAA AGG GAU AG) were designed. Temperature protocol for RT-PCR was 50 °C for 45 min, 94 °C for 2 min, 40 cycles consisting of 94 °C for 15 s, 53 °C for 30 s and 68 °C for 2 min and final extension of 68 °C for 7 min. Superscript^TM^ One-Step RT-PCR with Platinum Taq (Invitrogen, Life Technologies^TM^, Carlsbad, NM, USA) was used for the reaction mixture. Additionally nested step of the PCR (band size 653 bp) was carried out for some samples with previously published primers PPT 334C and PPT 986R [12]. To obtain PUUV M segment (band size 200 bp) previously published primers MOF 103, MOR 204, PUU F1 and PUU R1 were used [5,11].

#### 3.5.4. Tula Virus-Specific RT-PCR

To obtain TULV S segment (band size 653 bp) primers PPT 334C and PPT 986R [12] were used as previously described [6]. To obtain TULV M segment (band size 324 bp), primers MOF103, MOR204, PUU G1F and PUU G1R were used [11].

#### 3.5.5. Seewis Virus-Specific RT-PCR

To obtain SWSV SWSV-22-fw and SW-S-1590R primers for S segment (band size 1570 bp) [13] and OSV697, T-M1485R and M1199F primers for M segment (band size 250 bp) [14] were used as previously described [7].

### 3.6. Phylogenetic Analysis

All positive samples were sequenced with the ABI3500 Genetic Analyzer (Applied Biosystems, Life Technologies^TM^, Carlsbad, NM, USA). The obtained nucleotide sequences were analysed with CLC Main Workbench software, version 6.1 (CLC bio, Aarhus, Denmark). Sequence alignments were performed using the Muscle algorithm in MEGA version 5 [15]. Nucleotide substitution model (HKY + G) was selected based on Akaike’s information criterion (AIC) in jModelTest, version 0.1.1 [16]. Bayesian phylogenetic analysis was performed in MrBayes 3.2 [17] and Tracer version 1.5 [18]. Three independent MCMC runs of four chains each consisting of 10,000,000 generations were run to ensure effective sample sizes (ASS) of at least 200. Maximum clade credibility trees were depicted using FigTree version 1.3.1 [18]. Representative sequences of the hantavirus strains described in the study were deposited in GenBank (KF776554–KF776735, KF776736–KF776792, KF776793–KF776905).

### 3.7. Statistical Analysis

Statistical analysis was performed using IBM SPSS Statistics for Windows, Version 21.0. The relationship between phylogenetic clustering and zoogeographic regions was calculated using the χ^2^ test.

## 4. Conclusions

Slovenia lies at a junction of the Alps, the Mediterranean, the Pannonian basin and the Dinaric Mountains. The resulting high biotic and landscape diversity form the foundation for great variability of zoonotic pathogens, in our case the hantaviruses [8]. Thirty-one species of small mammals can be found in Slovenia [19,20]. These species occupy different habitats and also vary in their dietary preferences. Periodic fluctuation in rodent numbers is well recognized and several hypotheses have been offered to explain the pattern of inter-annual variation, from food availability to specialist predators or intrinsic factors [21]. Several species have been confirmed to harbour pathogenic (DOBV, DOBV–Kurkino and PUUV) and non-pathogenic (TULV and SWSV) hantaviruses [2,4,5,6,7,22]. DOBV and PUUV patients are confirmed annually in Slovenian HFRS-endemic regions, with a three-fold higher incidence of PUUV compared to DOBV. In the present study, we have genetically defined hantaviruses from one-fifth of Slovenian HFRS patients. Among them, the first patient with DOBV–Kurkino infection was discovered. Furthermore, several lineages have been recognized for each hantavirus species in Slovenia. The diversity within different hantavirus species ranges from 1.3% for DOBV–Kurkino lineages up to 14.1% for the SWSV (Figure 2). On average, higher nucleotide diversity was observed in non-pathogenic hantaviruses, whereby DOBV and PUUV seem to be more conserved, and with stronger geographical clustering. While no geographical clustering was observed for SWSV and two different phylogenetic lineages were simultaneously present in one sampling location, PUUV sequences cluster into three lineages, which are grouped in geographically limited areas (Figure 4). There is some overlap in the border areas between regions, where several genetic lineages are circulating. In comparison to previous studies, an additional genetic lineage (P1) of PUUV has been established in the Prealpine region, which has been severely affected during the last HFRS outbreak. Prior to the 2012 outbreak, only one PUUV RNA sequence obtained from a bank vole indicated the existence of this lineage and the endemic area. The regions where PUUV is established are rich in broad-leaved forests, which are favoured by PUUV rodent hosts. Bank voles prefer lowland forest habitats (plenty of beech and spruce), with major water flows in the vicinity. The genetic variability of the species has not been studied yet, but some variability in the species is recognized according to phenotype characteristics. The western part of the country, the Alpine and Dinaric regions, are settled by a bigger subspecies, most likely *M. glareolus gorka*, whereas the animals found in the Subpannonian region are somewhat smaller, though their subspecies is as of yet not recognized [20]. No connection can therefore be made between the subspecies distribution and the genetic variability of PUUV. A similarity can be observed in clustering of DOBV–Dobrava where again an overlap of several genetic lineages was observed in the border areas. Since most of the HFRS patients are infected in the vicinity of their home [4,5], the patient’s residence was used as a sequence geographic origin. The majority of patient sequences in the present study matched the host-derived sequences in the area, a fact which is therefore a good indicator that the infection originates locally, close to the patient’s home. Nevertheless, in the pyhlogeographic analysis of DOBV and PUUV, some outliers were present, which are derived from patient samples (Figure 4 and Figure 5). These patients were most likely infected outside of their local residence area; for example, during a vacation in a different region in Slovenia. An additional exception is a cluster of D3 lineage sequences, which have been detected in both mice and a patient in a limited, focal area in the Dinaric region, where the D2 lineage is prominent (Figure 5). In this focal area, it appears that both lineages circulate in rodents and occasionally patients are infected with either one or the other DOBV lineage. Although DOBV and PUUV are present in the majority of the country, the sequence diversity among DOBV lineages is lower in comparison to PUUV. The host, *A. flavicollis*, can be found all over Slovenia, but prefers dry and warm broad-leafed forests, especially in the Dinaric region. Variability of the species is low, and the animals probably belong to a single subspecies [20]. Additionally, the DOBV–Kurkino genotype has been confirmed both in a patient and striped field mice in the Subpannonian region. *A. agrarius* has a limited geographic range of distribution in Slovenia, and can be found only in parts of the Subpannonian region, a small area between the Subpannonian and Dinaric regions. An isolated population also exists in a restricted area in the Submediterranean region.

Hantavirus species are associated with a single rodent species in which they establish persistent infection [23]. DOBV and PUUV are both harboured by generalist rodent species, which can be found all over Slovenia. They are highly adaptable and able to adjust to almost any environment and may often survive habitat disturbances or even benefit from them. Both viruses can therefore be confirmed in a major part of the country. Contrary to these are the TULV, SWSV and DOBV–Kurkino genotypes. The *Microtus* voles, insectivores and the striped field mouse, which harbour these viruses, are specialist species. They are often significantly limited by the environment, due to their special dietary and habitat needs. Since their numbers are usually lower and there is less chance of exposure to humans, the human cases are less common. Both TULV and SWSV are probably not human pathogens and they have a high degree of nucleotide sequence divergence, which is often associated with hantaviruses in specialist species [24,25].

Due to the tectonic formation of particular areas and natural isolating barriers, climatic change and complex abiotic and biotic factors in a specific area, certain patterns of species distribution have been formed in Slovenia. These factors are included in the formation of the aforementioned zoogeographic regions designed by Mršić [8]. In our case, the influence of these factors is seen on the example of the species-specific hantaviruses. It is presumed that geographic barriers and the features of the zoogeographic area limit not only the virus host, but consequently also the virus, which can be found only within the limits (which are not always very strict) of specific zoogeographic areas. Within the zoogeographic regions, there are specific areas where hantavirus infections have been confirmed in the rodents and where human infections are most prevalent. These areas are most commonly associated with major water bodies in Slovenia, most often the major rivers (Figure 1). Most cities and villages in Slovenia are located close to the river systems, that is, the areas of highest diversity and species richness, which could thus explain why the majority of human cases appear in these regions. It is presumed that the combination of factors in endemic areas is such that it ensures a rodent population density which is above the threshold sufficient to ensure infection of the reservoir [26]. In years with a peak in rodent population, this threshold is achieved in a large part of the country, and hence the endemic regions seemingly explode due to dispersal of rodents from high-quality patches [27]. Still, geographic barriers seem to limit the spread of specific virus lineage and its host.

This study, which consists of one of the largest collections of hantaviral sequences to date, has shown a very high hantavirus diversity in a small area. It is evident that the distribution of the virus is mainly influenced by the presence of the host species, but the geographic characteristics impact and limit the formation of endemic areas. Specific features of a zoogeographic region should be analysed individually in order to recognize the most important factors influencing the hantavirus distribution. Previous studies recognized the importance of precipitation and landscape features (vegetation and soil variables) [27,28,29,30,31,32], which either influence the host ecology or intrinsic factors influencing virus transmission. Also, human influence should not be neglected in studies of endemic regions of hantaviruses. Previous studies confirmed that anthropogenic-disturbed land cover is associated with exposure of rodents to hantaviruses as well as the distribution and abundance of rodent species [33,34]. In the ever-changing environment, both in Slovenia and throughout the world, excellent knowledge of the variability of the hantavirus species, the recognition of endemic areas and important factors influencing those areas is essential. Such knowledge will enable us to make predictions of changes in disease risk in the future and consequently facilitate targeted intervention measures.
